# Aldosterone defects in infants and young children with hyperkalemia: A single center retrospective study

**DOI:** 10.3389/fped.2023.1092388

**Published:** 2023-01-16

**Authors:** Xu Liu, Yanshu Xie, Jing Tang, Jingzi Zhong, Dan Zeng, Dan Lan

**Affiliations:** Department of Pediatrics, First Affiliated Hospital of Guangxi Medical University, Nanning, China

**Keywords:** hyperkalemia, primary adrenal insufficiency, pseudohypoaldosteronism, aldosterone, novel mutation

## Abstract

**Introduction:**

Hyperkalemia is a rare but severe condition in young children and usually discovered as a result of hemolysis of the blood samples taken. However, patients with defects in either aldosterone biosynthesis or function can also present with hyperkalemia- as well hyponatremia-associated, and metabolic acidosis. It is a challenge to make an accurate diagnosis of these clinical conditions. We conducted this study to investigate the clinical and genetic features of aldosterone signaling defects associated hyperkalemia in young children.

**Method:**

A retrospective review was conducted at the pediatric department of the First Affiliated Hospital of Guangxi Medical University from 2012 to 2022.

**Results:**

47 patients with hyperkalemia were enrolled, of which 80.9% (*n* = 38) were diagnosed with primary hypoaldosteronism, including congenital adrenal hyperplasia due to 21-hydroxylase deficiency (*n* = 32), isolated hypoaldosteronism (*n* = 1) due to CYP11B2 gene mutation and Xp21 contiguous gene deletion syndrome (*n* = 1). Additionally, 4 patients were clinically-diagnosed with primary adrenal insufficiency. Nine patients were confirmed with aldosterone resistance, of which one child was diagnosed with pseudohypoaldosteronism (PHA) type 1 with a mutation in the NR3C2 gene and 3 children were identified with PHA type 2 due to novel mutations in either the CUL3 or KLHL3 genes. Five patients had PHA type 3 because of pathologies of either the urinary or intestinal tracts.

**Conclusions:**

The etiologies of infants with hyperkalemia associated with aldosterone defects were mostly due to primary hypoaldosteronism. An elevated plasma aldosterone level may be a useful biomarker for the diagnosis an aldosterone functional defect in patients presented with hyperkalemia. However, a normal plasma aldosterone level does rule out an aldosterone defect in either its biosynthesis or function, especially in young infants. Molecular genetic analyses can greatly help to clarify the complexity of disorders and can be used to confirm the diagnosis.

## Introduction

Potassium (K^+^) is the major electrolyte component in intracellular fluid (ICF) and is crucial for vital biological functions. Almost all of the total body K^+^ is located in the ICF, where it plays a crucial role in protein synthesis and cell growth (1, 2). A positive K^+^ balance is essential for somatic growth in growing children, especially in young infants. However, severe hyperkalemia is life-threatening and it can cause cardiac arrhythmias as well as cardiac arrest. Multiple etiologies can cause hyperkalemia including increased K^+^ load, renal dysfunction and certain drugs, but endocrine disorders should also be considered. Aldosterone (Ald) is the major mineralocorticoid hormone and it is a vital factor for maintaining electrolyte and water homeostasis in humans. It is produced exclusively in the adrenal zona glomerulosa and plays an important role in regulating the re-absorption of sodium (Na^+^) as well as the excretion of K^+^ *via* the distal nephrons (3). Ald secretion is mainly regulated by the renin–angiotensin system.

Ald defects, include impairments in Ald biosynthesis (primary hypoaldosteronism) and aldosterone resistance (pseudohypoaldosteronism). Defects in biosynthesis of Ald are usually related to genetic mutations which can result in a disability to produce enzymes or it can be caused by destruction or hypoplasia of adrenal cortex (4). Primary adrenal insufficiency (PAI) including congenital adrenal hyperplasia (CAH), X-linked adrenal hypoplasia congenita (X-AHC) and Xp21 contiguous gene deletion syndrome, are conditions characterized by a failure to produce cortisol and it is the most common etiology of Ald synthesis defects. Other etiologies such as isolated hypoaldosteronism (IHA) are rarely seen (5, 6). IHA, known as Ald synthase deficiency, is an extremely rare autosomal recessive disorder mainly caused by inactivating mutations of the *CYP11B2* gene (7, 8). Inactivating mutations in the *CYP11B2* gene can lead to abnormalities of aldosterone synthase which is the enzyme responsible for catalyzing the terminal steps in Ald biosynthesis and converts 11-deoxycorticosterone to Ald.

Ald resistance (9), is characterized by pseudohypoaldosteronism and result in insufficient K^+^ and hydrogen secretion (10, 11). Pseudohypoaldosteronism (PHA) has been described as three different forms: PHA types 1, 2 and 3 (PHA1, PHA2 and PHA3, respectively). PHA1 and PHA2 are referred to as primary pseudohypoaldosteronism. PHA1 is characterized by either abnormalities of the mineralocorticoid receptor or dysfunction in epithelial Na^+^ channel which responds to Ald in distal nephrons. Mutations in the mineralocorticoid receptor coding gene, *NR3C2*, and the epithelial Na^+^ channel coding gene of subunit genes (*SCNN1A*, *SCNN1B* and *SCNN1G*) are responsible for PHA1 (11).

PHA2 is known as familial hyperkalemic hypertension or Gordon's Syndrome. Mutations in the genes involved in either the pathway of the thiazide sensitive Na + chloride cotransporter (NCC) or renal outer medullary K^+^ channel (ROMK) are responsible for this disorder. The genes *WNK1*, *WNK4*, *KLHL3* and *CUL3* were shown to cause PHA2 (12–14). PHA3 (which is a secondary PHA or transient PHA), is caused by various pathologies of either the urinary and intestinal tracts or the sweat glands. Urinary tract infections and urinary malformations are the most common cause of PHA3 (15).

Patients with Ald defects typically present with growth retardation, salt-lost, hyperkalemia, hypovolemia and metabolic acidosis. However, rather than episodes of hypovolemia and salt-lost, patients with PHA2 usually present with low-renin hypertension, hyperkalemia and metabolic acidosis (12).

Numerous conditions such as CAH, IHA and PHA may present with similar clinical symptoms. This may result in difficulties producing a specific diagnosis for these conditions. The correct diagnosis is the most essential premise in order to achieve a successful treatment regimen. In this study, we present the clinical and genetic features of children with Ald signaling defects who are associated with hyperkalemia.

## Methods

### Patients and data collection

A retrospective study was conducted at the Pediatric unit of the First Affiliated Hospital of Guangxi Medical University from 1st January 2012 to 1st October 2022. The selection criteria were as follows: (1) clinical signs and symptoms associated with Ald defects, such as recurrent vomiting, growth retardation, hypertension in infants and young children (age under 3 years old). (2) hyperkalemia (serum K^+^ > 5.0 mmol/L) with or without hyponatremia (serum Na^+^ < 135.0 mmol/L) and metabolic acidosis. (3) If necessary, a positive genetic test relating to one of the etiologies of Ald defect by using an appropriate molecular genetic testing technology. Diagnosis of a disorder was based on its practical guidelines and for rare cases with no guidelines. This mainly depended on the clinical presentation, and results of biochemical examinations and molecular genetic testing. Patients with hyperkalemia but not due to aldosterone defects such as chronic kidney disease, hyperkalemia secondary to hemolysis, type IV renal tubular acidosis, medication related hyperkalemia were excluded from this study. Meanwhile, the patients with incomplete data were excluded from this study. The following data were collected from patients' records: demography, family history, clinical, biochemical, imaging tests and hormonal features at initial presentation. The results of genetic mutation analysis and treatments were also included.

## Results

Forty-seven patients with hyperkalemia were enrolled into this study. Of these, 38 patients were associated with Ald biosynthesis defects and 9 patients had related to Ald resistance.

### Patients with primary hypoaldosteronism

Thirty-eight patients were associated with defects in Ald biosynthesis and 32 of these contributed to CAH due to 21-hydroxylase deficiency. Four patients were clinically diagnosed with primary adrenal insufficiency with either a negative gene analysis or failure to conduct genetic testing. One patient was diagnosed with Xp21 contiguous gene deletion syndrome because of a rearrangement on Xp21 which contains the *NROB1* gene (Supplementary Table S1). All of them had hyperkalemia as well as hyponatremia.

An elevated level of serum adrenocorticotropic hormone (ACTH) combined with a low level of serum cortisol were observed in patients with primary adrenal insufficiency or Xp21 contiguous gene deletion syndrome. Test for hormone showed an elevated level of 17-hydroxyprogesterone (17-OHP) in all patients with CAH. Twenty-two patients with CAH were subjected to genetic analysis and all of them were shown to have mutations in the *CYP21A2* gene. In addition, one male infant was diagnosed with isolated hypoaldosteronism (IHA) due to a *CYP11B2* gene mutation and his detailed clinical features are shown in Table 1.

**Table 1:** Characteristics of young children with isolated hypoaldosterone and pseudohypoaldosteronism types 1 and 3.

Diagnosis	IHA	PHA					
		PHA1	PHA3	PHA3	PHA3	PHA3	PHA3
Age at diagnose	3 months	0.9 month	1.3 months	1.8 months	2.8 months	5.5 months	3 months
Sex	Male	Male	Female	Female	Female	Female	Male
Clinical symptom	Vomiting Poor weight	Fever	Fever diarrhea	Fever diarrhea vomiting	Poor weight	Poor weight	Irritability dyspnea convulsions
PE	Dehydration poor weight	Dehydration	Dehydration	Dehydration	Dehydration poor weight	Poor weight	Dehydration cyanosis
Plasma K (mmol/L)	6.6	7.31	8.23	6.1	6.53	6.3	5.97
Plasma Na (mmol/L)	124.7	125.7	114.3	127.6	119.4	125.3	125.3
Plasma PRA (ng/ml/h)	19.5	103.6	10.95	NA	14.05	NA	6.5
Plasma Ald (pg/ml)	65	2000	2000	3930.72	8785.66	2000	2074
Complications	None	Urinary tract infection	Intestinal tract infection	Intestinal tract infection	Urinary tract malformation and infection	Urinary tract malformation and infection	Urinary tract malformation and infection
Gene	CYP11B2	NR3C2	Negative	Negative	Negative	NA	NA
Mutation	c.1121C > T, c.1391_1393dupTGC	c.556_557del	NA	NA	NA	NA	NA
Reference	Data unpublished	Data unpublished	NA	NA	NA	NA	NA
Ongoing medications	FC	Oral sodium	None	None	None	Repeated antibiotics	None

IHA, isolated hypoaldosterone; PHA1, pseudohypoaldosteronism type 1; PHA3, pseudohypoaldosteronism type 3; PE, physical examination; K, potassium; Na, sodium; PRA, plasma rein activity; Ald, Aldosterone; NA, not available; FC, fludrocortisone; Plasma rein reference, 1.8–24.5 pg/ml; Plasma PRA, 0.93∼6.56 ng/ml/h; Plasma Ald reference, Infant 1–12 month 50–900 pg/ml.

### Patients with pseudohypoaldosteronism

All three types of PHA were all identified in our study, and one boy had PHA1 as well as a urinary tract infection. Five infants were associated with PHA3 which was secondary to the pathologies of either the urinary or intestinal tracts. Patients with either PHA1 or PHA3 all presented with similar clinical symptoms such as poor weight gain, vomiting and fever during infancy and their characteristics are shown in Table 1.

One infant and two young children were diagnosed with PHA2. They came to our hospital with different chief complaints and were incidentally discovered to have hyperkalemia and hypertension and they were finally diagnosed with PHA2. The first child was found to have a novel heterozygous mutation of the *KLHL3* gene, c.1484C > G (p.T495R), which was inherited from his father. The second child was observed to have a *de novo* mutation of the *CUL3* gene, c.560G > A (p.C187Y). The third infant was identified to have a novel pathogenic heterozygous mutation of the *KLHL3* gene, c.470_471delinsTG (p.A157V), which was inherited maternally. These variants had not been reported by either the ExAC or the Human Gene Mutation Database. Their clinical and genetic characteristics are shown in Table 2. Their family trees and Sanger sequencing are shown in Supplementary Figures S1–S3, respectively.

**Table 2:** Characteristics of patients with pseudohypoaldosteronism type 2.

Diagnosis	PHA2	PHA2	PHA2
Age at diagnosis	25 months	26 months	2 months
Sex	Male	Male	Male
Chief complaint	Severe hyperkalemia occurred during cleft lip and palate surgery	Speech delay	Erythema
PE	Hypertension	Hypertension growth retardation	Hypertension
Plasma K (mmol/L)	10.28	6.04	7.4
Plasma Na (mmol/L)	150.3	135.3	138
Plasma PRA (ng/ml/h)	3.68	10.46	NA
Plasma Ald (pg/ml)	141.9	137.1	NA
Gene	KLHL3	CUL3	KLHL3
Mutation	c.1484C > G (p.T495R)	c.560G > A (p.C187Y)	c.470_471delinsTG (p.A157V)
Inheritance	Father	De novo	Mother
Reference	This study	This study	This study
On-going medications	HCT	HCT	HCT

PHA2, pseudohypoaldosteronism type 2; PE, physical examination; K, potassium; Na, sodium; PRA, plasma rein activity; Ald, aldosterone; NA, not available; HCT, hydrochlorothiazide. Plasma PRA, 0.93∼6.56 ng/ml/h; Plasma Ald reference: Infant 1–12 month 50–900 pg/ml, children aged 2–10 years: 30–350 pg/ml.

## Discussion

Ald, a potent mineralocorticoid hormone, is synthesized exclusively by the adrenal zona glomerulosa. It can actively enhance Na^+^ reabsorption and secrete K^+^ and hydrogen ions *via* the distal renal nephrons. Ald biosynthesis defects or Ald resistance can result in electrolyte imbalance (hyponatremia with or without hyperkalemia) and metabolic acidosis. In most cases, hyponatremia can be easily identified, because it usually accompanied by some symptoms, such as recurrent vomiting, failure to thrive, dehydration and seizures. However, patients with hyperkalemia may be particularly difficult to recognize as they are often asymptomatic for most of the time. This is especially the case in young children. Severe hyperkalemia can be dangerous, if not identified and treated immediately. It may cause cardiac arrhythmias and arrest and can even lead to death. Hyperkalemia is a rare but severe condition in young children and in most cases, it is seen after difficulties in obtaining adequate blood samples due to hemolysis of these when being taken. However, patients with Ald signaling defects can also present with hyperkalemia and this may ignore by clinicians, especially when it occurs in young children.

Primary adrenal insufficiency, specifically to CAH with concomitant hypoaldosteronism is relatively common in young children. As in our study, this condition accounted for 68.1% (*n* = 32) of total cases with hyperkalemia (16). All of whom had hyperkalemia as well as hyponatremia during infancy. Steroid tests and genetic analysis can be useful to make a quick and correct diagnosis which can help when conducting genetic counseling to the prenatal mother. In addition to the most common cortisol deficiency associated with Ald defects (CAH due to 21-hydroxylase deficiency), several different etiologies related aldosterone signal defects were identified in this study including Xp21 contiguous gene deletion syndrome, IHA and PHA.

IHA is characterized by defects in Ald biosynthesis due to the Ald synthase deficiency. Most patients usually show a low plasma level of Ald. However, a normal or even a high level of plasma Ald was observed in some patients with IHA (17, 18). PHA1 is characterized by mineralocorticoid resistance due either to an inactive mutation in the mineralocorticoid receptor or the epithelial Na^+^ channel. Patients with PHA1 usually presented with a high level of serum Ald due to mineralocorticoid resistance. A marked elevated plasma Ald level may be a useful biomarker to distinguish the PHA1 from IHA. However, not all patients with PHA1 show elevated aldosterone levels (9). Actually, it is difficult to explain the circulating hormone levels observed during young childhood of these patients, especially in early infancy. If necessary, either repeated hormone testing or conducting serum and urine steroid precursors tests as well as performing a genetic analysis should be considered.

PHA2 is a rare hereditary disease and is associated with an over-activating thiazide-sensitive NaCl co-transporter in the distal nephrons, resulting in excessive Na^+^ resorption, which decreases the excretion of K^+^ and hydrogen (13, 19). It is characterized by hypertension, hyperkalemia, hyperchloremic metabolic acidosis and normal renal function. Plasma renin levels are usually suppressed and aldosterone levels can be variable but are relatively low due to the hyperkalemia (20). Blood pressure, which may be ignored in children, especially in early infancy, can be an important hint for establishing the diagnosis of PHA2. In our study, we carefully checked the blood pressure in all the young children and infants who presented with hyperkalemia without hyponatremia and finally we were able to make a correct diagnosis.

Ald is essential for K^+^ excretion and Na^+^ retention in the kidneys, salivary glands, sweat glands and colon. PHA3, known as reversible secondary pseudohypoaldosteronism, is described mainly because it is caused by urinary tract infections and anomalies during infancy. Watanabe et al. (21) reviewed 60 cases of secondary PHA and found all of them occurred at less than 7 months of age. In addition, all the patients suffered from urinary tract infections with or without urinary tract malformations. Patients with PHA3 due to pathology of the intestinal tract are relatively rare. Several patients who underwent operations for colectomy or small bowel resection or jejunostomy or ileostomy in adults and infants have been reported (22–24). However, patients with gastrointestinal losses-related PHA3 due to intestinal infections had been rarely reported. In our study, two infants who had not undergone any gastro-enteric operations were found to have PHA3 due to severe diarrhea associated with intestinal infections. Remarkably, it was difficult to distinguish PHA3 from PHA1 only by clinical symptoms and biochemical testing. This was particularly the case for patients with PHA1 who happened to have severe diarrhea or urinary tract infections, simultaneously. In our study, a male infant was found to have both urinary tract infection and PHA1. From our experience, imaging of the kidneys and adrenals, urinary analysis and tests for steroid precursors are recommended to exclude renal and adrenal causes. If the electrolyte disturbance cannot be corrected with the proper treatment towards primary disease in a relatively short time, molecular genetic analysis can be great helpful to make a correct diagnosis.

**Figure 1:**
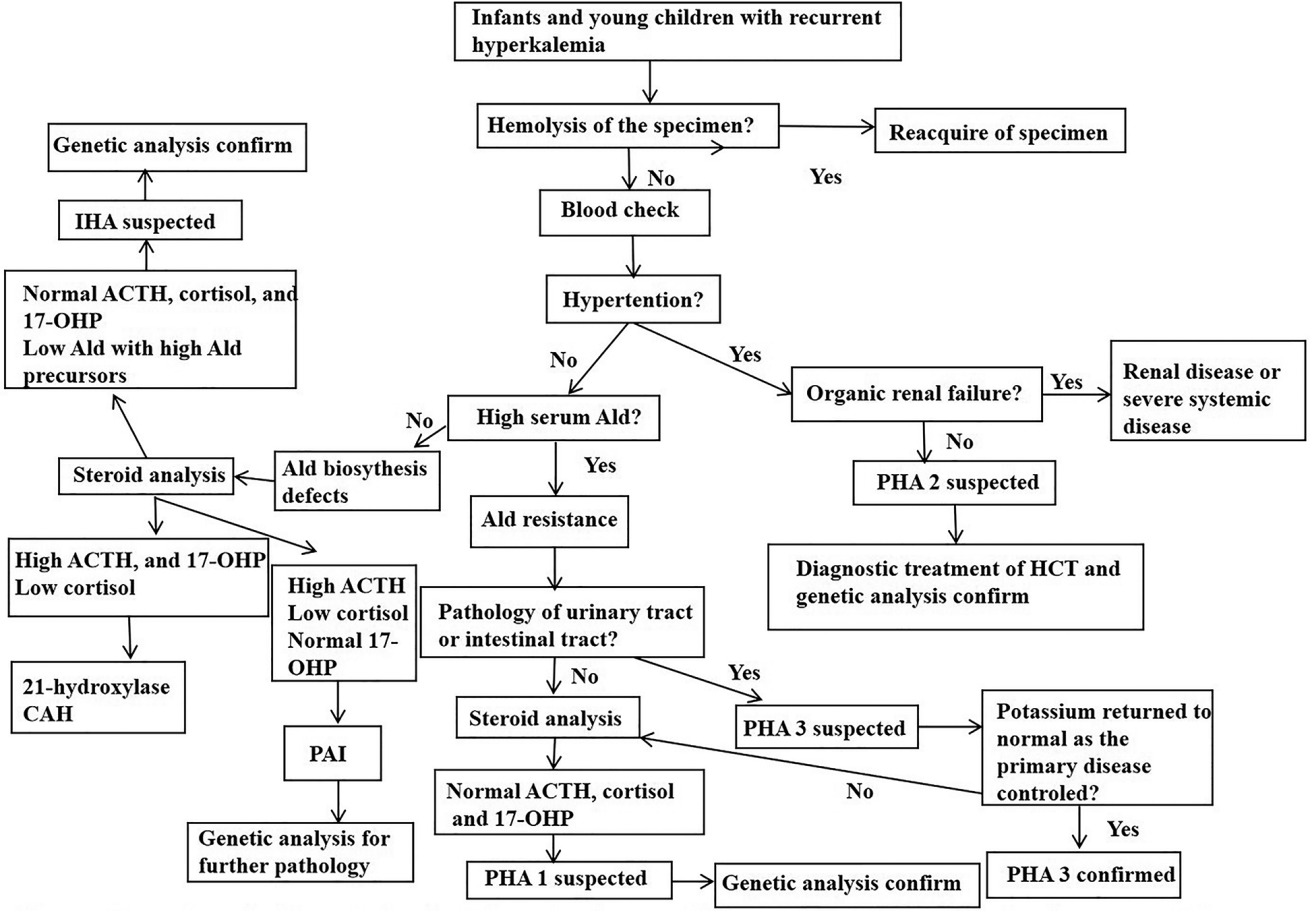
Proposed diagnostic procedure for inants and young children presenting with hyperkalemia who is suspected of aldosterone signal defect. Ald, aldoterone; CAH, congenital adrenal hyperplasia; PHA, pseudohypoaldosteronism; IHA, isolated hypoaldosterone; HCT, hydrochlorothiazide; 17-OHP, 17-hydroxyprogesterone.

CAH, PAI, IHA and PHA may all present with similar clinical symptoms which can make these conditions hard for a specific diagnosis. This study proposes a diagnostic procedure for infants and young children with hyperkalemia who are suspected with Ald signaling defects. This may be helpful for physicians to make a quick and correct diagnosis (Figure 1).

In this retrospective study, we also identified three novel heterozygous mutations of the *KLHL3* and *CUL3* genes which were associated with PHA2. Of these, there were two cases due to the *KLHL3* gene: c.1484C > G (p.T495R) and c.470_471delinsTG (p.A157V). The third was a *de novo* mutation of c.560G > A (p.C187Y) in the *CUL3* gene (Table 2).

The KLHL3 protein is thought to have three domains: a BACK (BTB and C-terminal Kelch) domain, a N-terminal BTB domain, and Kelch-like repeats forming a six-bladed *β*-propeller structure (12, 14, 25). The mutation of p.A157V is located in the BTB domain, which plays an important role in binding to the CUL3 protein. The p.T495R residue is located in the fifth Kelch motif of the protein and is involved in substrate binding. The clinical and genetic features of patients with mutations in *KLHL3* (19, 20, 26–34), including the present study and those who had clinical details published in previous reports, are summarized in Table 3. Heterozygous mutations are more common than homozygous ones. The age of the patients ranged from early infants to 69 years old. All of them had typical clinical symptoms including hyperkalemia and hypertension except for one 20-year-old female who had normal blood pressure. The mechanisms of the phenotype and genotype are still unclear. In our study, the two affected boys showed typical PHA2 presentation, but their 32- and 28-year-old father and mother both had the same *KLHL3* mutation, respectively, but were asymptomatic and had no hyperkalemia. More research and clinical details are needed to accurately and convincingly describe the genotype-phenotype correlation in this condition.

**Table 3:** Review of the characteristics of PHA2 patients with KLHL3 gene mutations.

Author	Age at diagnose (years)	Sex	Serum potassium	BP	Special features	Mutation	Exon	Het/Hom	Pattern inheritance
Holland (34)	0.33	F	8.6	105/58	None	R528H	13	Het	Dominant
Mayan (19)	69	F	5.4	115/78	None	Q309R	8	Het	Dominant
	48	F	5.2	147/95	None	Q309R	8	Het	Dominant
	11	M	6.4	104/61	Short stature	Q309R	8	Het	Dominant
	11	M	7.2	108/71	Short stature	Q309R	8	Het	Dominant
	10	F	5.3	103/72	None	Q309R	8	Het	Dominant
	36	F	5.6	154/77	None	R528H	13	Het	Dominant
	15	F	6.0	136/77	None	R528H	13	Het	Dominant
	13	F	6.2	124/77	None	R528H	13	Het	Dominant
	8	F	5.8	123/78	None	R528H	13	Het	Dominant
	2	M	7.0	146/95	Transient tachypnea of the newborn	R528H	13	Het	Dominant
Kelly (33)	18	M	7.3	150/90	None	H498Y	13	Het	Dominant
Mitani (20)	3	M	6.6	110/55	Short stature	L387P	9	Het	Dominant
Kliuk (32)	34	F	6.2	158/107	Toxemia of pregna on thiazi	S553L	13	Hom	Recessive
	26	M	7.1	115/70	Severe hyperkalemia when 1 year old	S553L	13	Hom	Recessive
	63	M	5.6	110/80	None	Q309R	8	Het	Dominant
	62	F	4.8	160/100	History of toxemia	Q309R	8	Het	Dominant
	57	M	5.1	120/90		Q309R	8	Het	Dominant
	54	M	6.4	145/105	Renal failure	Q309R	8	Het	Dominant
	47	F	5.1	120/90	History of toxemia	Q309R	8	Het	Dominant
	47	F	5.1	145/110	None	Q309R	8	Het	Dominant
	44	M	NA	160/100	None	Q309R	8	Het	Dominant
	18	M	5.2	110/70	None	Q309R	8	Het	Dominant
	15	F	5.8	110/80	None	Q309R	8	Het	Dominant
	12	F	5.3	114/56	None	Q309R	8	Het	Dominant
Park (31)	0.8	F	6.3	115/72	Poor oral intake growth retardation	C164F	4	Het	Dominant
	1.7	F	6.9	110/73	None	S433N	11	Het	Dominant
	23	F	6.4	130/85	None	S433N	11	Het	Dominant
Yang (29)	24	F	6.3	150/109	Episodic headache	S433N	11	Het	Dominant
Doan (30)	0.16	F	7.6	80/57	Poor weight gain	A474V	11	Het	Dominant
Anglani (35)	20	F	6.5	Normal	None	N529H	13	Het	Dominant
Zhang (28)	56	F	6.18	162/80	Hyperthyroidism	T110A	4	Hom	Recessive
Etges (26)	58	F	5.3	148/87	Chronic kidney disease	R431W	11	Hom	Recessive
This study	2.08	M	7.12	115/50	None	T495R	13	Het	Dominant
	0.25	M	7.4	110/65	Poor weight gain	A157V	5	Het	Dominant

F, female; M, male; BP, blood pressure; NA, not available; Het, heterozygous; Hom, Homozygous.

The *CUL3* gene is located in 2q36.2 and is also a causative gene for PHA2. To date, the reported PHA2-related CUL3 variants are all clustered in introns 8 and 9 and exon 9. These resulted in an in-frame 57 amino acid deletion of exon 9 (residues 403–459) which produced a truncated form of the CUL3 protein (14, 36). However, we found a young child who presented with typical symptoms of PHA2 carrying a *de novo* mutation of CUL3 in exon 5. This mutation was predicted to be pathogenic by bioinformatic tools including PolyPhen2, SIFT, Mutation Taster and ClinPred. According to the American College of Medical Genetics and Genomics guidelines (37), the *de novo* variant was classified as likely pathogenic. Remarkably, the CUL3 gene coding protein, is a component of a cullin–RING E3 ubiquitin ligase (38). It is involved in a wide range of critical cellular processes, including targeting proteins for proteasomal degradation, protein activity modulation, interaction and localization (14). CUL3 mutations may affect these activities, and therefore, would produce a very broad range of phenotypes. Recently, a CUL3 novel mutation was found in patients with autism (39). As with our study, the young child with the CUL3 gene mutation suffered from development delay and speech dyspraxia. The same condition was reported by Chatrathi et al. (40). We further summarized the patients with PHA2 due to the CUL3 gene mutations (34, 40–45) from the literature (Table 4).

**Table 4:** Review of the characteristics of PHA2 patients with CUL3 gene mutations.

Author	Age at diagnosis (years)	Sex	Serum potassium	BP	Special features	Mutation	Exon/intron	Het/Hom	Pattern inheritance
Tsuji (44)	3	F	6.8	124/30	None	c.1377G > C	E9	Het	Dominant
Osawa (45)	1	M	8.4	118/50	Growth retardation	c.382–6 T > G	intron 8	Het	Dominant
Hollander (34)	6	M	8.6	120/60	Hematuria	c.1377 + 1G > A	inron 9	Het	Dominant
Wang (41)	17	M	6.4	160/100	Short stature	c.1376 A > T	E9	Het	Dominant
Nakano (43)	0.16	F	High	Normal	None	c.1312A > G	E9	Het	Dominant
	2	M	Normal	Normal	None	c.1312A > G	E9	Het	Dominant
Park (42)	7	M	6.8	128/60	Short stature	c.1377 + 1G > C	intron 9	Het	Dominant
Chatrathi (40)	2.83	M	5.9	125/73	Global developmental delay Short stature	c.1420_1431del12	E 9	Het	Dominant
This study	2.5	M	5.81	120/90	Speech delay	c.560G > A	E5	Het	Dominant

F, female; M, male; BP, blood pressure; NA, not available; Het, heterozygous; Hom, Homozygous; E, exon.

Correct therapy aiming at the cause specific of a disease is needed to treat patients. For patients with PAI, glucocorticoids combined with fludrocortisone (FC) could get a good curative effect. Treatment with FC and oral sodium supplementation are essential for patients with mineralocorticoid hormone deficiency, especially when they are at a young age. Thiazide diuretics is an effective treatment for patients with PHA2. All patients with PHA2 in our study had normal blood pressure as well as normalized blood electrolyte when hydrochlorothiazide was administered as the therapy.

## Conclusions

In this retrospective study, we describe the etiologies of Ald signaling defects seen in our hospital in infants and young children who presented with hyperkalemia and we subsequently proposed a diagnostic procedure for these patients. We also identified 3 novel mutations in the *KLHL3* and *CUL3* genes which were associated with PHA2. These cases broaden the clinical and genetic correlations towards PHA2. This is the first study to contain a complete PHA cohort. However, as a retrospective study it has a number of limitations. More prospective and mechanistic studies are required in this field. Nonetheless, this has provided additional valuable clinical experience for diagnosing and treating patients with hyperkalemia due to Ald signaling defects.

## Data Availability

The original contributions presented in the study are included in the article, further inquiries can be directed to the corresponding author.
