# Supplementary material for: Aldosterone defects in infants and young children with hyperkalemia: A single center retrospective study
Source: Front Pediatr. 2023 Jan 16;11:1092388. doi: 10.3389/fped.2023.1092388 (PMC9885047; doi:10.3389/fped.2023.1092388)
Supplement: Supplementary file 1 [file Table1.docx]

Supplementary Material

Suplementary Fig. 1: Identification of mutation in KLHL3 gene in P1 wtih pseudohypoaldosteronism type2 (PHA2). A, The patient had a heterozygous mutation: c.1484C > G (p.T495R) inherited from his father. B, Pedigree of the family which shows the inheritance of the heterozygous mutation. The father(F) and the affected son (A) are represented by squares, and the mother(M) is represented by circle. The fathe is heterozygous and asymptomatic. The son inherited from his father but presented with hyperkalemia and hypertension.

**Suplementary Fig. 2:** Identification of mutation in CUL3 gene in P2 with PHA2. A, The patient had heterozygous mutation: c.560G > A (p.C187Y). B, Pedigree of the family shows the heterozygous muatation is a de novo mutation. F: father; M: mother; A: affected son.

**Suplementary Fig. 3:** Identification of mutation in KLHL3 gene in P3 wtih PHA2. A, The patient had a heterozygous mutation:c.470_471delinsTG (p.A157V) inherited from his mother. B, Pedigree of the familyh shows the inheritance of the heterozygous mutation. The mother (M) and the afected son (A) both have the heterozygous mutation, while, the mother is asymptomatic. F: father.

**Supplementary table.1:** Characteristics of infants and young children with CAH, PAI and Xp21 contiguous gene deletion syndrome

| No | Sex | Age at onset | Chief complaint | Clinical specific sign | K  (mmol/L) | Na  (mmol/L) | Diagnosis | Gene | Mutation |
| --- | --- | --- | --- | --- | --- | --- | --- | --- | --- |
| 1 | Male | 9 d | Poor feeding | None | 7.4 | 128.5 | 21-OHD CAH | CYP21A2 | c.713T>A  c.844G>T  c.955C>T |
| 2 | Female | 19 d | Fever | Hyperpigmentation  Virilization of external genitalia | 8.0 | 122 | 21-OHD CAH | CYP21A2 | c.332-339delGAGACTAC  c.710T>A c,713T>A c.844G>T c.1069C>T |
| 3 | Female | 1 m | Poor weight gain  Virilization of external genitalia | Hyperpigmentation  Virilization of external genitalia | 5.9 | 124.5 | 21-OHD CAH | CYP21A2 | c.293-13C>G  c.955C>T  c.1069C>T |
| 4 | Male | 2 m | Poor feeding  Poor weight gain | Hyperpigmentation | 5.6 | 120.5 | 21-OHD CAH | CYP21A2 | 293-13C>G  Rearrangement of 1-7 exon |
| 5 | Male | 15 d | Poor feeding  Poor weight gain | Hyperpigmentation | 7.8 | 118.3 | 21-OHD CAH | CYP21A2 | c.293-13C>G  c.1069C>T |
| 6 | Male | 1.8 m | Vomiting  Poor weight gain | Hyperpigmentation | 7.7 | 121 | 21-OHD CAH | CYP21A2 | Hom in c.293-13C>G |
| 7 | Male | 2 m | Vomiting  Poor weight gain | Hyperpigmentation | 7.0 | 103.5 | 21-OHD CAH | CYP21A2 | c.293-13C>G  c.844G>T  c.955C>T  c.1069C>T |
| 8 | Male | 2.5 m | Vomiting  Diarrhea  Poor weight gain | Hyperpigmentation | 6.1 | 115 | 21-OHD CAH | CYP21A2 | c.293-13C>G  c.518T>A  c.710T>A  c.713T>A  c.719T>A  c.1069C>CT  c.844G>T |
| 9 | Female | 10 d | Poor weight gain  Virilization of external genitalia | Hyperpigmentation  Virilization of external genitalia | 6.4 | 111.2 | 21-OHD CAH | CYP21A2 | c.713T>A  c.719T>A  c.1069C>T  c.651+37A>G |
| 11 | Male | 1.8 m | Vomting  Diarrhea  Poor weight gain | Hyperpigmentation | 7.6 | 105.5 | 21-OHD CAH | CYP21A2 | Hom in c.293-13C>G  Exon 1,3 large deletion |
| 12 | Female | 1.4 m | Poor weight gain  Virilization of external genitalia | Hyperpigmentation  Virilization of external genitalia | 7.4 | 110 | 21-OHD CAH | NA | NA |
| 13 | Female | 19 d | Virilization of external genitalia | Hyperpigmentation  Virilization of external genitalia | 6.4 | 129.6 | 21-OHD CAH | CYP21A2 | c.293-13C>G  c.518T>A |
| 14 | Male | 22 d | Vomting  Poor weight gain | Virilization of external genitalia | 10.7 | 104.1 | 21-OHD CAH | NA | NA |
| 15 | Male | 20 d | Poor weight gain | Hyperpigmentation | 7.2 | 117.9 | 21-OHD CAH | NA | NA |
| 16 | Male | 17 d | Poor weight gain | Hyperpigmentation | 8.8 | 109.3 | 21-OHD CAH | NA | NA |
| 17 | Male | 28 d | Poor weight gain  Vomiting | Hyperpigmentation | 5.86 | 105.2 | 21-OHD CAH | CYP21A2 | c.293-13C>G  Exon 1-7 large deletion |
| 18 | Female | 11 d | Fever  Virilization of external genitalia | Hyperpigmentation  Virilization of external genitalia | 6.5 | 128.6 | 21-OHD CAH | CYP21A2 | Hom in c.293-13C>G |
| 19 | Female | 13 d | Vomiting  Virilization of external genitalia | Hyperpigmentation  Virilization of external genitalia | 8.9 | 110.4 | 21-OHD CAH | CYP21A2 | c.293-13C>G  c.923dupT  Exon 1,3,4,6,7 large deletion |
| 20 | Male | 15 d | Vomiting | Hyperpigmentation | 6.4 | 127.9 | 21-OHD CAH | NA | NA |
| 21 | Female | 4 m | Virilization of external genitalia | Hyperpigmentation  Virilization of external genitalia | 5.6 | 132.1 | 21-OHD CAH | CYP21A2 | c.293-13C>G  c.518T>A |
| 22 | Female | 4 m | Virilization of external genitalia | Hyperpigmentation  Virilization of external genitalia | 5.3 | 133.5 | 21-OHD CAH | CYP21A2 | c.293-13C>G  c.518T>A |
| 23 | Male | 28 d | High 17-OH progesterone in newborn screening | Hyperpigmentation | 6.3 | 120 | 21-OHD CAH | CYP21A2 | c.293-13C>G  c.1451G>C  c.1455delG |
| 24 | Female | 1 m | Virilization of external genitalia  Diarrhea | Hyperpigmentation  Virilization of external genitalia | 6.4 | 121 | 21-OHD CAH | NA | NA |
| 25 | Male | 21 d | Fever  Vomiting | Hyperpigmentation | 6.5 | 94.7 | 21-OHD CAH | CYP21A2 | Hom in c.293-13C>G |
| 26 | Male | 3 m | Vomiting  Poor weight gain | Hyperpigmentation | 6.3 | 122.5 | 21-OHD CAH | NA | NA |
| 27 | Male | 2 m | Poor weight gain | Hyperpigmentation | 6.14 | 128.5 | 21-OHD CAH | NA | NA |
| 28 | Female | 1 m | Virilization of external genitalia | Hyperpigmentation  Virilization of external genitalia | 6.4 | 119.8 | 21-OHD CAH | CYP21A2 | c.293-13C>G  c.518T>A  c.552C>G |
| 29 | Male | 2 m | Vomiting  Poor weight gain | Hyperpigmentation | 6.0 | 102 | 21-OHD CAH | CYP21A2 | Hom in c.293-13C>G |
| 30 | Female | 15 d | Vomiting  Virilization of external genitalia | Hyperpigmentation  Virilization of external genitalia | 6.5 | 121 | 21-OHD CAH | CYP21A2 | c.293-13C>G  c.1481G>A |
| 31 | Female | 1.9 m | Virilization of external genitalia | Hyperpigmentation  Virilization of external genitalia | 6.6 | 119 | 21-OHD CAH | CYP21A2 | c.713T>TA  c.651+37A>G  c.293-67C>A |
| 32 | Male | 2 m | Vomiting  Poor weight gain | Hyperpigmentation | 6.14 | 132 | 21-OHD CAH | NA | NA |
| 33 | Male | 1.5 m | Vomiting  Poor weight gain | Hyperpigmentation | 6.0 | 123.4 | PAI | Negative | Negative |
| 34 | Male | 4 m | Vomiting  Poor weight gain | Hyperpigmentation | 5.6 | 125.8 | PAI | Negative | Negative |
| 35 | Male | 3 y | Vomiting  Fever | Hyperpigmentation | 7.84 | 110.4 | PAI | NA | NA |
| 36 | Male | 3 m | Vomiting  Poor weight gain | Hyperpigmentation | 6.5 | 124 | PAI | NA | NA |
| 37 | Male | 3 y | Trouble walking  Weakness | Hyperpigmentation | 5.04 | 126 | Xp21 contiguous gene deletion syndrome | Xp21.1  rearrangement  contains NROB1 | Xp21.1  rearrangement  contains NROB1 |

PE: physical examination; d: days; m: months; y: years. FC: fludrocortisone; HC: hydrocortisone; CAH: congenital adrenal hyperplasia;

PAI: rimary adrenal insufficiency; 21-hydroxylase deficiency: 21-OHD; hom: homozygous; NA: not available.

**Supplementary table.1:** Characteristics of infants and young children with CAH, PAI and Xp21 contiguous gene deletion syndrome

| No | Sex | Age at onset | Chief complaint | Clinical specific sign | K  (mmol/L) | Na  (mmol/L) | Diagnosis | Gene | Mutation |
| --- | --- | --- | --- | --- | --- | --- | --- | --- | --- |
| 1 | Male | 9 d | Poor feeding | None | 7.4 | 128.5 | 21-OHD CAH | CYP21A2 | c.713T>A  c.844G>T  c.955C>T |
| 2 | Female | 19 d | Fever | Hyperpigmentation  Virilization of external genitalia | 8.0 | 122 | 21-OHD CAH | CYP21A2 | c.332-339delGAGACTAC  c.710T>A c,713T>A c.844G>T c.1069C>T |
| 3 | Female | 1 m | Poor weight gain  Virilization of external genitalia | Hyperpigmentation  Virilization of external genitalia | 5.9 | 124.5 | 21-OHD CAH | CYP21A2 | c.293-13C>G  c.955C>T  c.1069C>T |
| 4 | Male | 2 m | Poor feeding  Poor weight gain | Hyperpigmentation | 5.6 | 120.5 | 21-OHD CAH | CYP21A2 | 293-13C>G  Rearrangement of 1-7 exon |
| 5 | Male | 15 d | Poor feeding  Poor weight gain | Hyperpigmentation | 7.8 | 118.3 | 21-OHD CAH | CYP21A2 | c.293-13C>G  c.1069C>T |
| 6 | Male | 1.8 m | Vomiting  Poor weight gain | Hyperpigmentation | 7.7 | 121 | 21-OHD CAH | CYP21A2 | Hom in c.293-13C>G |
| 7 | Male | 2 m | Vomiting  Poor weight gain | Hyperpigmentation | 7.0 | 103.5 | 21-OHD CAH | CYP21A2 | c.293-13C>G  c.844G>T  c.955C>T  c.1069C>T |
| 8 | Male | 2.5 m | Vomiting  Diarrhea  Poor weight gain | Hyperpigmentation | 6.1 | 115 | 21-OHD CAH | CYP21A2 | c.293-13C>G  c.518T>A  c.710T>A  c.713T>A  c.719T>A  c.1069C>CT  c.844G>T |
| 9 | Female | 10 d | Poor weight gain  Virilization of external genitalia | Hyperpigmentation  Virilization of external genitalia | 6.4 | 111.2 | 21-OHD CAH | CYP21A2 | c.713T>A  c.719T>A  c.1069C>T  c.651+37A>G |
| 11 | Male | 1.8 m | Vomting  Diarrhea  Poor weight gain | Hyperpigmentation | 7.6 | 105.5 | 21-OHD CAH | CYP21A2 | Hom in c.293-13C>G  Exon 1,3 large deletion |
| 12 | Female | 1.4 m | Poor weight gain  Virilization of external genitalia | Hyperpigmentation  Virilization of external genitalia | 7.4 | 110 | 21-OHD CAH | NA | NA |
| 13 | Female | 19 d | Virilization of external genitalia | Hyperpigmentation  Virilization of external genitalia | 6.4 | 129.6 | 21-OHD CAH | CYP21A2 | c.293-13C>G  c.518T>A |
| 14 | Male | 22 d | Vomting  Poor weight gain | Virilization of external genitalia | 10.7 | 104.1 | 21-OHD CAH | NA | NA |
| 15 | Male | 20 d | Poor weight gain | Hyperpigmentation | 7.2 | 117.9 | 21-OHD CAH | NA | NA |
| 16 | Male | 17 d | Poor weight gain | Hyperpigmentation | 8.8 | 109.3 | 21-OHD CAH | NA | NA |
| 17 | Male | 28 d | Poor weight gain  Vomiting | Hyperpigmentation | 5.86 | 105.2 | 21-OHD CAH | CYP21A2 | c.293-13C>G  Exon 1-7 large deletion |
| 18 | Female | 11 d | Fever  Virilization of external genitalia | Hyperpigmentation  Virilization of external genitalia | 6.5 | 128.6 | 21-OHD CAH | CYP21A2 | Hom in c.293-13C>G |
| 19 | Female | 13 d | Vomiting  Virilization of external genitalia | Hyperpigmentation  Virilization of external genitalia | 8.9 | 110.4 | 21-OHD CAH | CYP21A2 | c.293-13C>G  c.923dupT  Exon 1,3,4,6,7 large deletion |
| 20 | Male | 15 d | Vomiting | Hyperpigmentation | 6.4 | 127.9 | 21-OHD CAH | NA | NA |
| 21 | Female | 4 m | Virilization of external genitalia | Hyperpigmentation  Virilization of external genitalia | 5.6 | 132.1 | 21-OHD CAH | CYP21A2 | c.293-13C>G  c.518T>A |
| 22 | Female | 4 m | Virilization of external genitalia | Hyperpigmentation  Virilization of external genitalia | 5.3 | 133.5 | 21-OHD CAH | CYP21A2 | c.293-13C>G  c.518T>A |
| 23 | Male | 28 d | High 17-OH progesterone in newborn screening | Hyperpigmentation | 6.3 | 120 | 21-OHD CAH | CYP21A2 | c.293-13C>G  c.1451G>C  c.1455delG |
| 24 | Female | 1 m | Virilization of external genitalia  Diarrhea | Hyperpigmentation  Virilization of external genitalia | 6.4 | 121 | 21-OHD CAH | NA | NA |
| 25 | Male | 21 d | Fever  Vomiting | Hyperpigmentation | 6.5 | 94.7 | 21-OHD CAH | CYP21A2 | Hom in c.293-13C>G |
| 26 | Male | 3 m | Vomiting  Poor weight gain | Hyperpigmentation | 6.3 | 122.5 | 21-OHD CAH | NA | NA |
| 27 | Male | 2 m | Poor weight gain | Hyperpigmentation | 6.14 | 128.5 | 21-OHD CAH | NA | NA |
| 28 | Female | 1 m | Virilization of external genitalia | Hyperpigmentation  Virilization of external genitalia | 6.4 | 119.8 | 21-OHD CAH | CYP21A2 | c.293-13C>G  c.518T>A  c.552C>G |
| 29 | Male | 2 m | Vomiting  Poor weight gain | Hyperpigmentation | 6.0 | 102 | 21-OHD CAH | CYP21A2 | Hom in c.293-13C>G |
| 30 | Female | 15 d | Vomiting  Virilization of external genitalia | Hyperpigmentation  Virilization of external genitalia | 6.5 | 121 | 21-OHD CAH | CYP21A2 | c.293-13C>G  c.1481G>A |
| 31 | Female | 1.9 m | Virilization of external genitalia | Hyperpigmentation  Virilization of external genitalia | 6.6 | 119 | 21-OHD CAH | CYP21A2 | c.713T>TA  c.651+37A>G  c.293-67C>A |
| 32 | Male | 2 m | Vomiting  Poor weight gain | Hyperpigmentation | 6.14 | 132 | 21-OHD CAH | NA | NA |
| 33 | Male | 1.5 m | Vomiting  Poor weight gain | Hyperpigmentation | 6.0 | 123.4 | PAI | Negative | Negative |
| 34 | Male | 4 m | Vomiting  Poor weight gain | Hyperpigmentation | 5.6 | 125.8 | PAI | Negative | Negative |
| 35 | Male | 3 y | Vomiting  Fever | Hyperpigmentation | 7.84 | 110.4 | PAI | NA | NA |
| 36 | Male | 3 m | Vomiting  Poor weight gain | Hyperpigmentation | 6.5 | 124 | PAI | NA | NA |
| 37 | Male | 3 y | Trouble walking  Weakness | Hyperpigmentation | 5.04 | 126 | Xp21 contiguous gene deletion syndrome | Xp21.1  rearrangement  contains NROB1 | Xp21.1  rearrangement  contains NROB1 |

PE: physical examination; d: days; m: months; y: years; FC: fludrocortisone; HC: hydrocortisone; CAH: congenital adrenal hyperplasia;

PAI: primary adrenal insufficiency; 21-hydroxylase deficiency: 21-OHD; hom: homozygous; NA: not available.
