# Supplementary figures and images for: Aldosterone defects in infants and young children with hyperkalemia: A single center retrospective study
Source: Front Pediatr. 2023 Jan 16;11:1092388. doi: 10.3389/fped.2023.1092388 (PMC9885047; doi:10.3389/fped.2023.1092388)

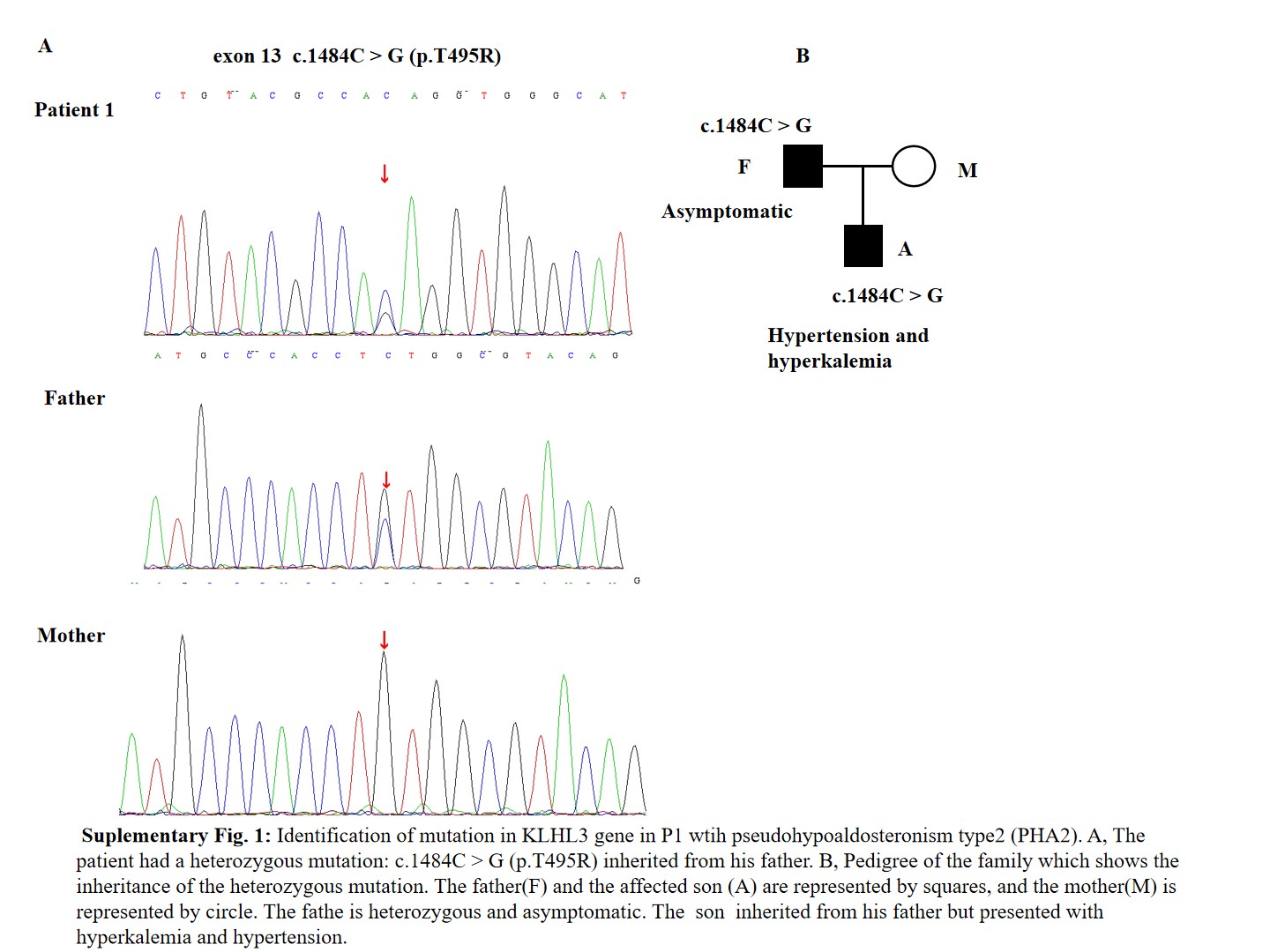

Supplement: Supplementary file 2 [file Image1.jpeg]

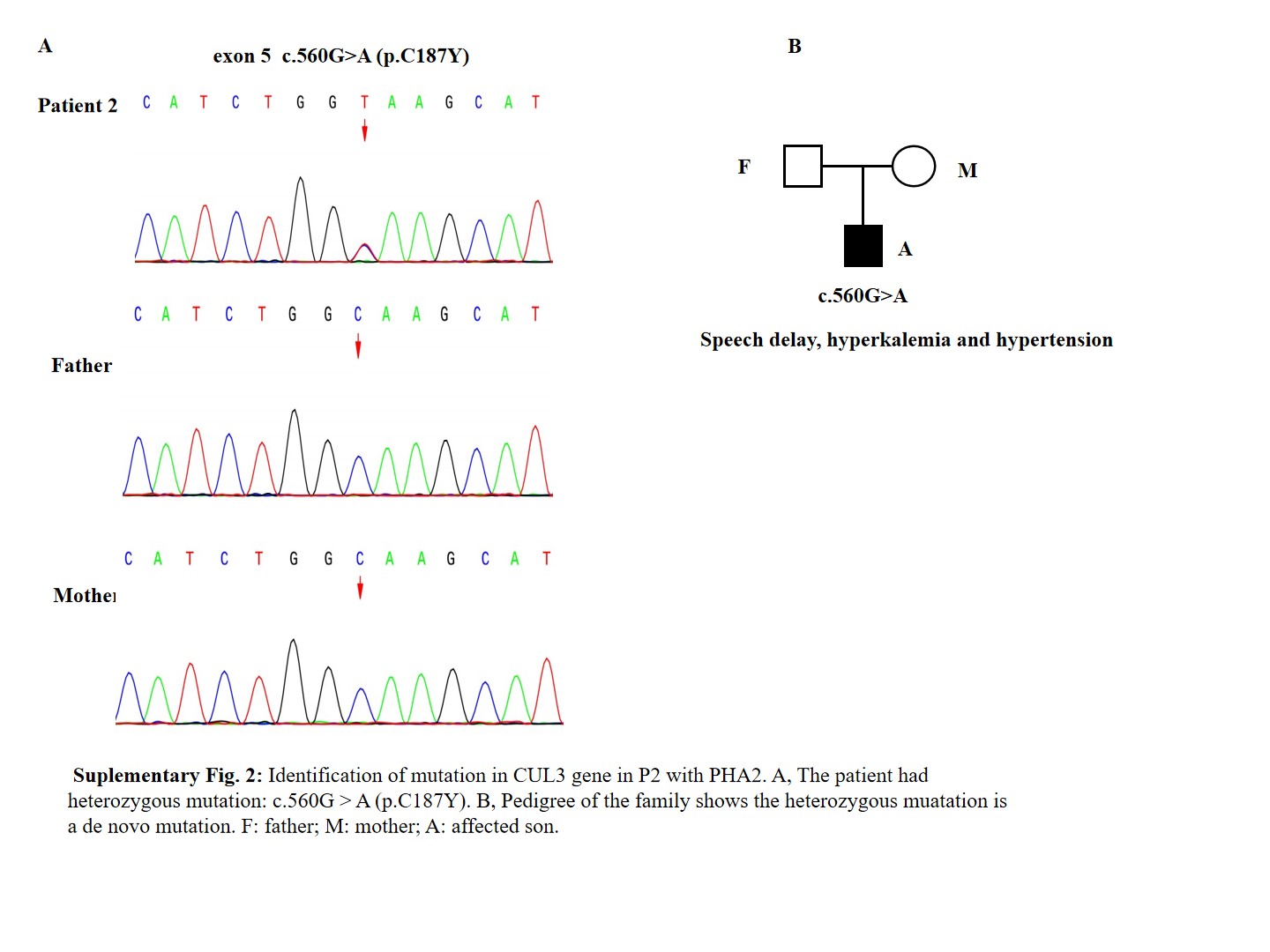

Supplement: Supplementary file 3 [file Image2.jpeg]

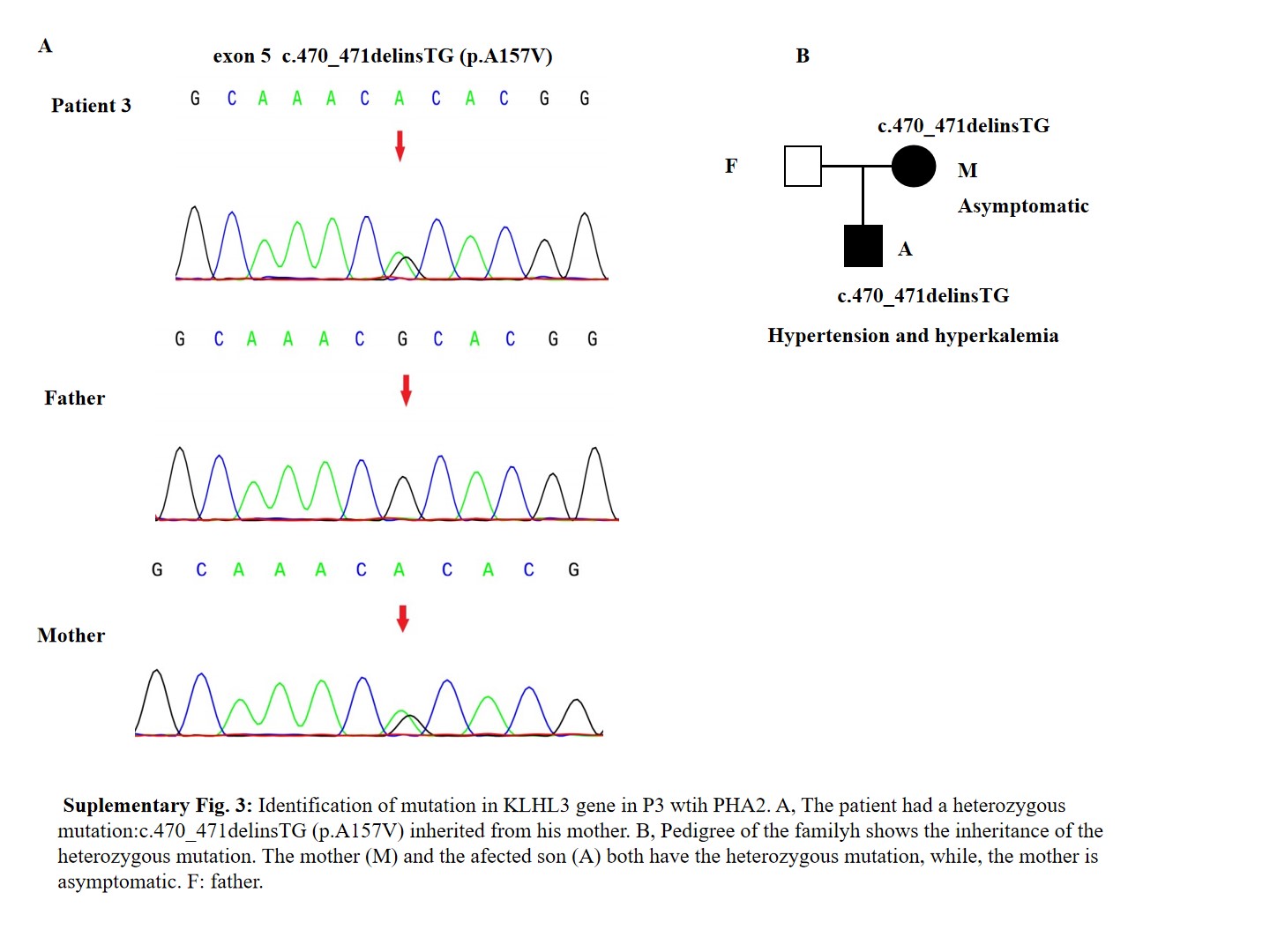

Supplement: Supplementary file 4 [file Image3.jpeg]
